# Extent of East-African Nurse Leaders' Participation in Health Policy Development

**DOI:** 10.1155/2012/504697

**Published:** 2012-10-04

**Authors:** N. Shariff, E. Potgieter

**Affiliations:** ^1^Advanced Nursing Studies Programme, The Aga Khan University, P.O. Box 39340, Nairobi 00623, Kenya; ^2^Department of Health Studies, University of South Africa (UNISA), P.O. Box 392, Pretoria 0003, South Africa

## Abstract

This paper reports part of a bigger study whose aim was to develop an empowerment model that could be used to enhance nurse leaders' participation in health policy development. A Delphi survey was applied which included the following criteria: expert panelists, iterative rounds, statistical analysis, and consensus building. The expert panelists were purposively selected and included national nurse leaders in leadership positions at the nursing professional associations, nursing regulatory bodies, ministries of health, and universities in East Africa. The study was conducted in three iterative rounds. The results reported here were gathered as part of the first round of the study and that examined the extent of nurse leaders' participation in health policy development. Seventy-eight (78) expert panelists were invited to participate in the study, and the response rate was 47%. Data collection was done with the use of a self-report questionnaire. Data analysis was done by use of SPSS and descriptive statistics were examined. The findings indicated that nurse leaders participate in health policy development though participation is limited and not consistent across all the stages of health policy development. The recommendations from the findings are that health policy development process needs to be pluralistic and inclusive of all nurse leaders practicing in positions related to policy development and the process must be open to their ideas and suggestions.

## 1. Introduction and Background Information

The World Health Organization (WHO) in the 49th World Health Assembly (WHA 49) recognized the potential of nursing to make a major contribution regarding the quality and effectiveness of health services. It suggested that nurses and midwives must be involved at all levels of the health systems and urged member states to involve nurses in health care policy and reform [1]. Since then, the agenda of strengthening nursing and midwifery has remained an important and recurring item of the WHO assemblies. 

In 2003, the World Health Assembly (WHA 56/19) recognized that in order to achieve the Millennium Development Goals (MDGs), there is a need to provide support for countries to strengthen their nursing and midwifery services [2]. WHO has acknowledged that up to 90% of the health workforce comprises of nurses. Nurses and midwives make substantial contribution to health-delivery systems in primary care, acute care, and community care settings [3]. Despite their contribution to health care, they are seldom involved in policy development [3]. Still more worrying is that, according to WHO [2], nurses' input into health policy development appears to be decreasing. It is suggested that in order to include nurses in the health policy debate, governments must develop legal frameworks to ensure clear nursing representation [2]. 

Whilst nurse legends such as Florence Nightingale and Lilian Wald were prominent in influencing policy development, this tradition was not sustained by contemporary nurses and interest in health policy has only recently reemerged [4]. In 2000, the International Council of Nurses (ICN) adopted the position that nurses have an important contribution to make in health services planning and decision making and in development of appropriate and effective health policy. Nurses can and should contribute to public policy pertaining to the determinants of health. The ICN's viewpoint on the urgency of nurses' involvement in health policy development was affirmed in 2005 when a document on “guidelines on shaping effective health policy” was issued [5, page 19]. Hennessy [6] contends that the shape of health care provision and the health of the population are subject to nurses' input in health policy and hence the latter must participate in the policy development process. 

Lima and Sampaio [7] affirm that there was slight political movement in the 1940s, but it was not until the 1970s and 1980s that nurses began serious political activities to influence health policy in the developed countries. Although nurses from western countries such as the USA and UK have made significant progress in influencing health policy development, they still face significant challenges. This is true for nurses even when they are part of the government system. For example, in a qualitative study conducted by Dollinger in the USA [8] that examined nurses' influence in health policy, the findings revealed that nurses who work in the government have limited ability to influence policy due to the negative image and status accorded to the nursing profession and the dominance of the medical profession in policy development. 

Kunaviktikul et al. [9] explored the knowledge and involvement of hospital-based clinical nurses and national nurse leaders in health policy in Thailand. Their findings revealed that the majority of the former were not involved in national health policy development. Nurse leaders who were involved in the policy development process were mainly part of the policy implementation stage and were not involved in policy formulation or even modification stages. 

In a unique African study, Phaladze [10] investigated the role of nurses in the human immunodeficiency virus/acquired immune deficiency syndrome (HIV/AIDS) health policy process in Botswana. The sample included policy makers and nurse leaders. The findings indicated that the majority of the nurses were aware of the national HIV/AIDS policy, but only a small minority was involved in the policy development process; this was mainly during the policy adoption, implementation, and evaluation stages. It was found that whilst the policy makers acknowledged that the omission of nurses from the policy development process was a mistake, they ironically blamed the nurses for not being proactive towards issues related to HIV/AIDS. On the other hand, the policy makers did not feel that nurses possessed the competence to participate in policy decisions. Furthermore, they also blamed the Ministry of Health (MOH) for not including nurses in the process and acknowledged that doctors were dominant [10]. 

The studies examined indicated that nurses' role in the health policy development process is limited. And when they are included in the policy development process, nurses are largely expected to be part of the policy adoption and implementation process. The image and status of nursing influences nurses inclusion in the policy development process. 

In East Africa, nurse leaders' role in health policy development is unclear. Literature searches revealed very limited information related to nurses and nurse leaders' participation in the health policy development process, particularly from the context of the developing world, and especially East Africa. Nurses' role in health policy development needs to be strengthened; however, it can only be reinforced if the phenomenon is studied and understood. The purpose of this part of the study was to explore the extent of nurse leaders' participation in health policy development in East Africa. The knowledge gained from the study informed the development of an empowerment model that may enhance nurse leaders' participation in this field.

## 2. Research Methodology

### 2.1. Research Design

A Delphi survey was applied and it included the following criteria: expert panelists, iterative rounds, statistical analysis, and consensus building. The Delphi survey was applied as it has been utilized to develop models and frameworks [11]. 

The study was conducted in the three East African countries of Kenya, Uganda, and Tanzania. The target population for this study comprised of nurse leaders who occupy national or provincial leadership positions in East Africa. The sample was derived from the target population consisted of nurse leaders working in national or provincial leadership positions from the Ministry of Health (or equivalent), nursing councils, national nurses associations, and universities. 

Purposive sampling was used because the intention was to include participants who were knowledgeable about the subject being studied. The sampling framework developed for the study required that the sample fulfill the following criteria. (1) Be a registered nurse; (2) Currently working in a senior leadership/management capacity in East Africa; (3) Currently working at national/provincial (regional) level or at a university. Additionally, the “closeness continuum” developed by Needham and De Loë [12, page 138] was applied. As per the criteria proposed in the “closeness continuum,” nurse leaders with subjective expertise, mandated expertise, and objective expertise were included in the study. Subjective expertise included possessing knowledge in terms of health policy implementation or affected by health policy or who may participate in the health policy development process. For the purpose of this study, they were provincial nurses/public health nurses, leaders in national nurses' professional associations who also work as registered nurses. Mandated expertise entailed the knowledge and experience in terms of the job requirement related to participation in health policy development process. For the purpose of this study, they were chief nurses/registrars/chairpersons of nursing regulatory bodies/national nurses' professional associations leaders/deputies. Objective expertise entailed knowledge gained through academic position, education and research with regard to policy development process. For the purpose of this study, they were academic nurse leaders/deans/academic heads. 

The study provided an opportunity for 78 nurse leaders from the three East African countries to be part of the study and in this part of the study, 37 (47%) responded. The study was conducted in three iterative rounds, and this paper reports findings from the first round.

### 2.2. Questionnaire Development

The data collection tool was developed by the researchers. The tool was a questionnaire and was developed with reference to research literature. The questionnaire included two sections. *Section  1* covered demographic data on the panelists with reference to country represented, organization represented, number of years of experience in nursing, and number of years in current position. The demographic data helped to confirm that the sample was representative of nurse leaders as proposed in the sampling framework and possessed the critical characteristics relevant to achieving the aim of the study. *Section  2* aimed to answer the objectives of the study. The first objective aimed to “explore the extent of nurse leaders' participation in health policy development” was explored and is reported in this paper. Published literature influenced the conceptualization of these questions in the questionnaire. Consequently, questions included in the questionnaire related to stating the major components of their current job positions and membership of nursing professional organizations and were influenced by Hayes and Fritsch [13] who report that membership of a professional organization was associated with political participation. The questions seeking to understand nurses' participation in policy development were influenced by the model developed by Cohen et al. [14, pages 259, 260] representing the “Stages of Nursing's Political Development.” The questions related to the extent of participation in the stages of the policy development process was influenced by the model of policy development which includes problem identification and agenda setting, policy formulation, policy implementation, and policy evaluation [15]. 

Pretesting of the questionnaires was conducted. The participants who were selected to pretest the tool comprised a purposive sample of nurse leaders (senior management level positions whose role included participation in the policy development process). These nurse leaders worked in a private regional hospital (1), district hospitals (2), a national military referral hospital (3), and as senior lecturers (4). The participants who were included in the pretesting of the questionnaires were excluded from the main study. The criteria for pretesting the questionnaires were length, clarity, language, relevance, overall adequacy, and whether content reflected health policy development and nurses' participation in health policy development. 

The participants found the questions clear, language acceptable, topic relevant to nursing, and related to health policy development and the instrument user friendly. 

### 2.3. Validity

Delphi surveys are mainly concerned with face validity and content validity. 

#### 2.3.1. Face Validity

Refers to whether the instrument *looks *as though it is measuring the appropriate concepts [16]. It includes the questionnaire being readable, exhibiting clarity of content, language, and being unambiguous [17]. As reported in the above section, the tool was pre-tested to ensure face validity. 

#### 2.3.2. Content Validity 

Refers to the choice of items and questions that are being explored and examined [18]. In this study, content validity was enhanced by firstly referring to the literature related to health policy development and nurses' participation in health policy development. Secondly, the questionnaire was pretested with a representative sample of nurse leaders, to ensure that the concepts included in the study were actually related to health policy development process. Thirdly, utilization of a purposive sample of a panel of experts that participate in the health policy development process. 

### 2.4. Data Analysis

The computer package SPSS (Statistical Package for Social Scientists, version 15) was utilized. Data was analyzed utilizing descriptive statistics. Ascertaining the groups' collective opinion required the use of descriptive statistics, which were carried out in this study in consultation with a statistician [19]. Descriptive statistics describe data utilizing numerical and graphical summaries [20]. The descriptive statistics utilized included univariate analysis of the data into frequency distribution. Frequency distributions summarize and compress data by grouping them into classes and recording how many data points fall into each class [20]. Data reported in this paper is mainly frequency distribution that includes summaries of categories and percentages. 

### 2.5. Ethical Considerations

Ethical clearance was obtained from the Health Studies Research and Ethics Committee of the College of Human Sciences, University of South Africa (reference number/project number 3273 404 2). Approvals were secured from the National Council for Science and Technology of Kenya (reference number-NCST/5/002/R/427), National Institute for Medical Research of Tanzania (reference number/NIMR/HQ/R.8a/Vol.IX/904), and Uganda National Council for Science and Technology (reference number/698/07/1). 

The *right to autonomy was respected *and informed consent was attained by explaining the benefits, rights, and risks involved in the research study in writing. Consent to participate was assumed by the return of the questionnaire.

*Confidentiality *was maintained at all times throughout the study's data collection phase. Once the questionnaires were returned, they were kept under lock and key to ensure that apart from the researchers no one else had access to the information. The participants expressed their opinions, anonymously. The information collected was presented anonymously as group views. This meant that the data presented in aggregate form represents the collective views of the expert panel members [21].

### 2.6. Data Collection Process

A database with the current information pertaining to the nurse leaders was unavailable. Therefore a database of expert panelists was created by calling the relevant offices and seeking the information related to the expert panelists. This formed the basis on which the sampling framework was developed. The expert panelists were telephonically informed that a questionnaire related to the study was on the way and that their participation would be appreciated. The questionnaires were delivered to all the 78 nurse leaders incorporated in the study via email, and hard copies were hand delivered and also sent to their postal addresses with self-addressed envelopes to facilitate return of the questionnaire. Follow-up reminders were made via email and by telephone. The data collection process took six weeks and began on 22 September 2009 up to 30 October 2009, for the first round. 

## 3. Results

The results presented here include the demographic data; major components of job position in relation to policy development; membership and role in professional organization; ways of involvement in health policy development; views on nurses' participation in health policy development; nurse leaders' participation in policy development at global, regional, national, and provincial levels; nurse leaders' participation in the stages of the policy development process. 

### 3.1. Demographic Data

#### 3.1.1. Country and Setting

The majority of the expert panelists who participated in the study were from Kenya 16 (43.2%), followed by Tanzania 15 (40.6%) and Uganda 6 (16.2%). Higher numbers were from urban settings, 21 (57%) (Table 1). 

#### 3.1.2. Organization

The majority of the expert panelists in the first round were from the ministry of health 17 (45.9%) or academic organizations 10 (27%) (Table 1).

#### 3.1.3. Age and Gender

The majority of the expert panelists were above 40 years of age: 33 (89%) and 24 (65%) were over 51 years old (Table 1). The majority of the expert panelists 23 (62.2%) were females. However, these percentages do not reflect the proportion of males/females in nursing in East Africa; for example, in Kenya, the percentage of males in nursing is about 28% [22]. 

#### 3.1.4. Education

The majority of the expert panelists 26 (70%) possessed a university degree (Table 1). Whilst there is scant literature on the demographic attributes of national nurse leaders, Carroll's [23] study conducted in the USA revealed that all the nurse executives in her study had earned a minimum of a master's degree. 

#### 3.1.5. Experience

The majority of the expert panel members, 31 (86%), reported over 15 years of experience in nursing. Out of these, 54% recorded more than 25 years of experience. This indicates that the majority of the expert panelists had considerable experience and expertise in the nursing profession [24]. Almost three quarters, 27 (73%), of the expert panelists reported up to five (5) years of experience in their current position. This indicates that, whilst the majority of the expert panelists 31 (86%) reported over 15 years of experience in nursing, only over a quarter (27%) had 6 to 15 years of experience in their current position. This may indicate that nurse leaders do not remain in health policy positions for long periods, by the time they secure policy related positions they are older (as noted in Table 1) and nursing might be losing nurse leaders with experience in health policy to retirement.

### 3.2. Major Components of Job Position in relation to Policy Development

The nurse leaders identified three major roles as management which included planning, supervision, monitoring, staffing, and resource management; education which included teaching and training; policy development which included participation in policy formulation and policy implementation as well as interpretation and dissemination of policy. The roles related to policy development were related mainly to policy formulation and implementation and did not include the whole policy development process. Deschaine and Schaffer [25] report similar policy development responsibilities among public health nurse leaders in the USA. In contrast their study did not report that nurses have educational or policy dissemination roles as was seen in the current study. 

### 3.3. Membership and Role in Professional Organization

The majority of the expert panelists were part of a professional association: 35 (94.6%). Two (5.4%) left this question blank, perhaps indicating that they were not members of any such organization. The major role that was identified by the expert panelists in the professional organization was that of being members. A few identified themselves as playing leadership roles in the organization. Their role in terms of influencing health policy development or political activism through their professional organization was unclear. Membership in itself does give nurses an opportunity to be more politically active and to participate in health policy development. Two separate studies by Hayes and Fritsch [13] and Small [26] conducted in the USA, among registered nurses, found a relationship between membership of a professional organization and political participation. The latter is linked to the ability to influence the course of health policy development. Professional organizations provide the opportunity for political participation. 

### 3.4. Ways of Involvement in Health Policy Development

The expert panelists indicated that they become involved in health policy development through nursing organizations, position(s) held, and on an individual basis. Camunas [27] and Gebbie et al. [28] concur with these findings and they further include community, government, and educational institutions as spheres of nurses' influence on health policy. 

### 3.5. Views on Nurses' Participation in Health Policy Development

The findings suggest that in the perception of the expert panelists, nurses' involvement in the policy arena is limited. In other words, the wider profession of nursing beyond those in formal leadership positions appear to have a less significant role in policy development. It is significant that the health policy agenda proposed at policy forums is largely dictated by others, not nurses themselves, despite their comprising the largest health workforce (Table 2). To have nurses' concerns and issues recognized as health policy priorities, it is important that all nurses be actively involved in influencing the health policy agenda [14]. There is need for a bottom up approach to health policy development. 

### 3.6. Nurse Leaders' Participation in Policy Development at Global, Regional, National, and Provincial Levels

**Figure 1:**
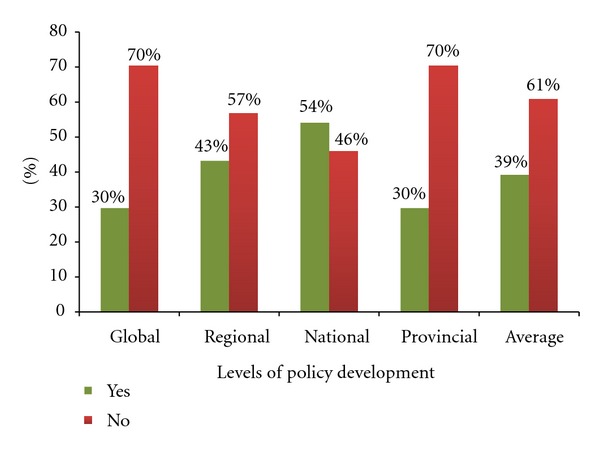
Participation in policy development at global, regional, national, and Provincial levels (*n* = 37).

Over half of the expert panelists participate in health policy development at national level: 20 (54%). However, their participation decreases at regional 16 (43%), global 11 (30%), and provincial 11 (30%) levels of health policy development (Figure 1). 

It is important to note that currently the East African countries are establishing the East African Community; these findings suggest that whilst some of the nurse leaders are involved in regional health policy development processes, there is an opportunity for securing a higher degree of involvement.

### 3.7. Nurse Leaders' Participation in the Stages of the Policy Development Process

**Figure 2:**
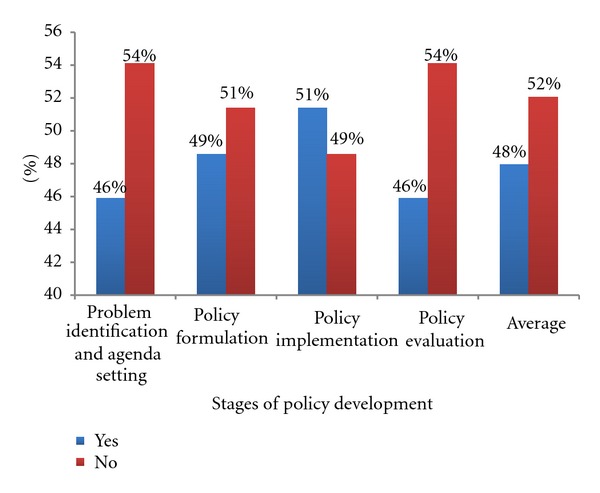
Participation in stages of the policy development process (*n* = 37).

Over half 20 (51%) of the expert panelists do participate in health policy implementation. However, their participation decreases at other stages of the process, for instance, in problem identification 17 (46%), policy formulation 18 (49%), and evaluation 17 (46%). The findings here indicate that the expert panelists do participate in this process, however not throughout the process and not consistently; nevertheless nursing is present to some degree in the health policy development process. Additionally, they may be part of the process on an ad hoc basis (Figure 2).

## 4. Discussion

The findings of this study indicate that nurse leaders participate in the health policy development process to some extent. More nurse leaders participate at national levels of health policy development compared to the provincial, regional, and global levels. Fewer nurse leaders participate throughout the health policy development process. Their input was the greatest at the policy implementation stage. The results also indicate that a significant proportion of nurse leaders are not part of the policy development process. This suggests that the process may not be sufficiently pluralist and inclusive for all nurse leaders to participate in health policy development. Nurse leaders' jobs do require them to be part of the health policy development process at the level of policy formulation and policy implementation although their role is largely managerial and administrative. Whilst the majority of the expert panelists belonged to their professional organization, their role was limited to being members. There is significant support from nurse leaders for professional nursing organizations in terms of membership. Professional organizations appear to be underutilized as a vehicle for political activism to influence health policy development with regard to nurses' concerns in health care. Conversely, their ability to be politically active might be restricted due to the nature of their job positions. 

## 5. Recommendations

This study indicates that there is a window of opportunity for nurses and nurse leaders' to enhance their participation in health policy development nationally and regionally. This has implications on nursing practice and nursing research.

### 5.1. Nursing Practice

The findings of this study indicated that there is only a small contribution from nurse leaders in health policy development. Their participation in health policy development consequently needs to be enhanced. The health policy development process needs to be pluralistic and inclusive of all nurse leaders practicing in positions related to policy development, and the process must be open to their ideas and suggestions. The opportunity to participate needs to be made available at all stages of the health policy development process to all nurse leaders currently in leadership positions and the wider nursing fraternity. Nurse leaders who are currently in health policy development positions need to create opportunities to enhance nurses' participation in terms of the increasing number of nurses who take part in the process. Being included will enable nurses to gain experiential knowledge, exposure, and confidence in the field of health policy development. 

### 5.2. Nursing Research

This is a formative study that is unique in the sense that it explores the gap in literature related to nurse leaders' participation in health policy, encompassing the extent of participation from the East African perspective. Further studies need to be conducted on the journey and career pathways of nurse leaders who are able to actively participate and contribute effectively in health policy development, so that nurses who want to make health policy a career can learn from their success and challenges. Research needs to be conducted with other levels of nurses to understand their knowledge, practice, and attitudes towards participation in health policy development and what measures can be taken to enhance their participation. 

## 6. Limitations of the Study

The study was conducted in the three East African countries of Kenya, Tanzania, and Uganda. Other countries in East Africa were omitted. Therefore, the findings are only applicable to the countries where the study was conducted. The sample was a purposive sample and was selected as per the researchers' knowledge of the contribution that the expert panelists could make to the study. This may have resulted in some relevant nurse leaders being excluded and that may have potentially influenced the results of the study otherwise.

## 7. Conclusion

The study is unique in the African context and particularly in the Kenyan, Ugandan, and Tanzanian perspective, where little is known about the extent of nurse leaders' participation in health policy development because the phenomenon appears to be underexplored, as noted in current literature. This study affords an indication of the extent of their actual participation in this regard. The results indicate that nurse leaders do participate in health policy development, however, this is limited to some nurse leaders only and is not uniform throughout the process. The results indicate that there is a window of opportunity for nurse leaders to enhance participation in health policy development process.

## Figures and Tables

**Table 1:** Demographic data (*n* = 37).

		Number	Percentage
Countries	Kenya	16	43.2%
	Tanzania	15	40.5%
	Uganda	6	16.2%

Setting	Urban centers	21	57%
	Rural centers	16	43%

Organizations	Nursing regulatory bodies	6	16.2%
	Ministry of Health (or equivalent) (CNO/deputy CNO, provincial matrons)	17	45.9%
	National nurses' professional associations	4	10.8%
	Universities	10	27%

Age	>40 years	33	89%
	<40 years	4	11%

Gender	Female	23	62%
	Male	14	38%

Education	Degree	26	70%
	Diploma	11	30%

Years of experience in nursing	>15 years	32	86%
	<15 years	5	14%

Years of experience in current position	>5 years	10	27%
	Up to 5 years	27	73%

**Table 2:** Views on nurses' participation in health policy development.

	Number	Percentage
Individual nurses *do not *participate in *health policy development *	25	68%
Nurses *do not* participate in *nursing related health policy* development	19	51%
Nurses *do not* participate on *broader health policy development* issues	22	59%
Nurses *do not lead* in *setting *the health policy development *agenda *	34	92%
